# Nutritional Description of Processed Foods with Fibre-Related Nutrition Claims in Spain: The BADALI Project

**DOI:** 10.3390/nu15163656

**Published:** 2023-08-20

**Authors:** Ana B. Ropero, Fernando Borrás, Marta Rodríguez, Marta Beltrá

**Affiliations:** 1 Institute of Bioengineering, Miguel Hernández University, 03202 Elche, Spain; beltra@umh.es; 2 Department of Statistics, Mathematics and Informatics, Miguel Hernández University, 03202 Elche, Spain; f.borras@umh.es; 3 Polytechnic School of Orihuela, Miguel Hernández University, 03202 Elche, Spain; marta.rodriguez11@goumh.umh.es

**Keywords:** nutrient composition, nutrition claims, fibre, nutrition claims, nutrient profile/profiling model, fortification, sweeteners, healthy food, food database, plant-based meat analogues

## Abstract

Fibre is one of the most beneficial nutrients for health and is very frequently used in nutrition claims (NCs) to promote foods. These claims may lead consumers to believe that products bearing them are healthy and/or healthier than those without them. The main objective of this work is to address this belief. This is the first exhaustive analysis of seven processed food types with fibre-related NCs (six cereal-based and one plant-based meat analogues) comparing them with those without these claims. The Spanish Food Database, BADALI, was used for this study. Results show that as many as 88.7% of processed foods with fibre-related NCs are classified as ‘less healthy’ according to the Nutrient Profile Model developed by the Pan American Health Organization (PAHO-NPM). When compared to foods without these NCs, similar results were obtained in the whole sample. Most of the observed divergences when analysing individual critical nutrients by food type indicate a deterioration of the nutritional quality. Foods with fibre-related NCs contained more fibre. The more frequent use of whole grain cereals or other fibre-specific ingredients may contribute to this. Some other nutritionally relevant differences were observed and half of them reflected a deterioration of the nutritional quality. In addition, these foods presented a lower prevalence of the organic version, as well as similar rates of mineral and vitamin fortification. Therefore, processed foods with fibre-related NCs are not healthy, nor present a better nutritional profile than those without.

## 1. Introduction

Dietary fibres ‘include any edible parts of the plant or analogous carbohydrates that are resistant to digestion in the small intestine and fermented in the large intestine’ [1]. According to the Global Burden Disease Study, 606.220 deaths in the world were attributable to diets low in fibre in 2019 [2]. As Mathers states, ‘there is now convincing evidence from prospective cohort studies that diets low in dietary fibre are associated with increased risk of common non-communicable diseases including CVD, type 2 diabetes and colorectal cancer’ [3]. A high intake of dietary fibre has also been associated with reduced risk of other types of cancer, such as oesophageal, gastric, breast, endometrial ovarian, renal cell, prostate and pancreatic cancer [4]. 

Dietary fibre is a myriad of different compounds, such as beta-glucan, pectin, resistant starch and many others. Whether individual compounds have the same health effects is a matter of study. For example, evidence indicates that oat beta-glucan reduces serum cholesterol and postprandial glycaemic responses [5]. ‘Individuals’ postprandial blood glucose and leptin responses were reduced after consuming RS, lowering the risk of insulin and leptin resistance’ [6]. 

Fibre and its benefits are frequently used to draw consumers’ attention to foods, for example, by using nutrition and health claims (NHCs) [7,8,9,10,11,12,13,14]. Many works have shown that NHCs may increase the perceived nutritional quality and healthiness of foods as well as influence purchasing behavior [15,16]. However, these results have been challenged by others showing no effect or even decreasing purchase intentions of foods with NHCs [15]. Moreover, the presence of NCs may reduce the probability of understanding information about food composition [17]. 

Similar conflicting results have been obtained when studying consumers’ perception of fibre-related NCs. On one hand, some works indicate positive attitudes. A study conducted in the USA showed that ‘high fibre’ was one of the nutrient content claims contributing to the overall judgement of foods as healthy [18]. Another work showed that ‘source of fibre’ was the most important characteristic on the product package in order to select a biscuit among all tested [19]. Additionally, participants perceived high fibre content as being beneficial to health [19]. In an Italian study, one third of consumers rated NCs on fibre to be ‘very’ or ‘extremely’ interesting [20]. A study in Chile showed that these claims used in breakfast cereals led to more positive ratings compared to fat-related claims or the absence of any claim at all [21]. An oat fibre claim that is applied to breakfast cereals resulted in more positive attitudes among Canadian consumers compared to the taste control claim [22]. It was also more influential on purchasing intentions and consumers believed that products were healthier [22]. On the other hand, some works failed to show a positive influence of fibre-related NHCs on consumers. As an example, the use of NCs on fibre in cereal bars did not influence the healthiness perceived by Uruguayan consumers [23]. 

Despite these contradictory results, some evidence suggests that fibre-related NCs of foods may affect consumers’ perception about their nutritional quality. Whether these foods are any better than those without these claims has been addressed by three studies in Italy and Canada [12,13,14]. The one in Canada analysed a great variety of products and the authors applied a nutrient profile model to determine their nutritional quality [12]. However, the nutrient composition was not examined [12]. The two Italian works investigated the nutrient composition of plant-based meat analogues and breakfast cereals [13,14]. Therefore, a complete nutritional study of a greater variety of products bearing fibre-related claims as well as a comparative analysis with those without these claims has not been published yet. 

Fibre-related NCs are usually intended to potentiate the perceived healthiness of foods. Another tool to make them more appealing to consumers is nutrient fortification [24,25]. So far, no publication has been released investigating the nutrient fortification of foods bearing fibre-related NCs. The organic origin is another factor influencing the alleged healthiness of foods [26,27]. The interaction between fibre-related NCs and the organic claim has only been studied once, and the work made reference to breakfast cereals [14]. 

Therefore, this is the first exhaustive analysis of processed foods bearing fibre-related NCs comparing them with those without these claims in large samples of seven specific food types. It includes the study of both the nutrient composition and nutrition quality by applying a nutrient profile model. It also incorporates the interaction between the use of fibre-related NCs and nutrient fortification (fibre, vitamins and minerals), as well as the prevalence of organic foods.

## 2. Materials and Methods

### 2.1. BADALI Database of Processed Foods for Sale in the Spanish Market

The authors elaborated the food database BADALI at Miguel Hernández University in 2016 as a social project to assist citizens to improve their diets [28]. BADALI includes nutritional information about thousands of foods for sale in the Spanish market. Information such as nutrient composition, health and nutrition claims is regularly collected and updated, preferentially from manufacturers’ websites, but also from online supermarkets. The presence of the nutrition declaration in the food information is required in order to be included in BADALI. The updated version of the database can be consulted online at https://badali.umh.es (accessed on 30 June 2023) [29]. 

At present, the food database is used to carry out studies on nutrition, food science and public health as previously published [7,30,31,32,33]. Foods analysed in the present study were gathered from June 2022 to June 2023. According to Ropero et al., 2023, cereals had the highest prevalence of fibre-related NCs, while their presence in other food types was minor [7]. Therefore, cereal-based processed foods were selected for this study (Table 1). The presence of bars made with non-cereal ingredients, such as nuts, legumes and fruit, is increasingly common. Since consumers may consider them as similar to those made with cereals, they were included in this food type. In addition, plant-based meat substitutes were detected to use fibre-related NCs quite frequently. Consequently, they were analysed in the present study (Table 1).

Before starting this study, the database was checked for inconsistent data, which were excluded from the analysis. In addition, when two or more foods differed only in size, one was preserved and the rest were discarded. Some foods did not display the energy content. In these cases, it was calculated using the following coefficients: 4 kcal/g protein and carbohydrate, 9 kcal/g total fat; 2.4 kcal/g polyols and 2 kcal/g fibre [34]. To list a product as organic, food information must include the international symbol or any of the related words (eco, ecologic, bio, organic).

### 2.2. Nutrition Claim (NC) Analysis

Nutrition claims (NCs) were analysed following previous publications [7,30,31]. Information provided by the online supermarkets was very limited regarding these claims. Therefore, in order to avoid bias, only the main food image provided was checked for NCs. Clear images of some products could not be obtained and, thus, they were excluded from the present work. In order to avoid any misinterpretation, only text NCs were registered. 

Foods were classified into two categories based on the absence or presence of fibre-related NCs. There are only two specific NCs on fibre authorised by the EU. To claim that a product is a ‘source of fibre’, a minimum of 3 g fibre/100 or 1.5 g fibre/100 kcal is required [35]. ‘High fibre’ may only be used when the product contains at least 6 g fibre/100 g or 3 g fibre/100 kcal [35]. One additional NC was also registered: ‘more fibre’, which is included in the authorised NC ‘increased’ [35]. 

Some wording flexibility rules were required following Ropero et al. and Regulation (EC) No 1924/2006 [7,35]:

- Rich in/very rich/excellent/large source of/important source of fibre means ‘high fibre’;
- The word ‘fibre’ anywhere in the image was interpreted as ‘source of fibre’;
- More fibre means ‘increased’ fibre.

### 2.3. Nutrient Composition of Foods and ‘Healthiness’ Evaluation

The nutrient composition of the two food categories was compared using statistics. To determine nutritionally relevant differences, the criteria previously used in Ropero et al., 2023, were followed [31]. This was based on the conditions applying to the NCs ‘increased’ and ‘reduced’ included in the Annex of the European Regulation (EC) N° 1924/2006 [35]. Therefore, a 30% higher median value was required to consider that processed foods with fibre-related NCs presented increased contents of any nutrient and energy [35]. Conversely, a 30% decrease was necessary to consider a relevant reduction for all nutrients, except 25% for sodium [35]. 

The Nutrient Profile Model developed by the Pan American Health Organization (PAHO-NPM) was utilised to classify foods as ‘healthy’ or ‘less healthy’ as previously published [31,32,36,37]. Processed foods are considered ‘less healthy’ when reaching or exceeding any threshold for total fat, saturated fat, free sugar or sodium. The PAHO-NPM also includes a threshold for trans fat, which could not be used due to lack of data. This model considers foods with any low- or no-calorie sweeteners (LNCSs, including polyols) as ‘less healthy’, regardless of their functionality (as sweeteners or any other use) [36]. Thresholds stablished by the PAHO-NPM are: (1) ≥30% of total energy from total fat, (2) ≥10% of total energy from saturated fat, (3) ≥10% of total energy from free sugars and (4) ≥1 mg sodium/kcal [36]. Only foods with data for all five components (total fat, saturated fat, free sugar, sodium/salt, LNCS) were included in the global statistics for ‘less healthy’. The PAHO-NPM was used throughout the entire sample regardless of the degree of processing, because it is a research work.

Ropero et al., 2023, was followed to estimate free sugar content in cereal-based foods [31]: (1) for those with no added sugar, free sugar was 0; (2) for the rest, 2 g sugar/100 g was subtracted from total sugar because this is the naturally occurring sugar content in most frequently consumed grains in Spain [38]. For plant-based meat analogues and bars made with non-cereal ingredients (not included in ref. [31]), the free sugar definition developed by Public Health England published in 2018 was used, specifically Table 1 of Swan et al. [39]. 

### 2.4. Fortification with Fibre, Vitamins and Minerals

Ingredient lists were checked for the presence of any whole grain cereal and fibre-specific ingredients. The inclusion criteria for the latter were as follows: (1) the presence of any ingredient known to be fibre or used mainly to provide fibre and (2) the absence of any indication of an additive function for this ingredient. Detected fibre-specific ingredients were: acacia fibre, agar-agar, amylopectin, apple fibre, bamboo fibre, barley fibre, beetroot fibre, beta-glucan, bran, buckwheat fibre, carob fibre, carrageenans, carrot fibre, cellulose, citrus fibre, corn fibre, flaxseed fibre, FOS, GOS, hemicellulose, hydroxypropylmethylcellolose, inulin, isomaltooligosaccharide, isomaltulose, konjac fibre, modified starch, mucilages, oat fibre, pea fibre, pectin, pineapple fibre, polydextrose, potato fibre, psyllium (plantago fibre, plantago psyllium), resistant dextrin, resistant starch, rice fibre, rye fibre, soy fibre, tapioca soluble fibre, vegetable fibre, wheat fibre and xantan.

The inclusion criteria included in Ropero et al., 2023, were followed to register vitamin/mineral fortification [31]: (1) a chemical providing a vitamin/mineral listed as ingredient and (2) the absence of any indication of an additive function for this chemical. A chemical containing two minerals was registered as fortified with both. As an example, the addition of potassium chloride was recorded as fortified with potassium and chloride. The alga Lithothamnium calcareum was listed as calcium fortification. 

### 2.5. Statistics

The Kruskal–Wallis H test, commonly referred to as the ‘one-way ANOVA on ranks’, is a valuable nonparametric statistical test utilised to investigate significant differences between multiple food groups, each corresponding to an independent variable and a continuous or ordinal dependent variable. Unlike parametric ANOVA, this test does not make assumptions regarding the normality of random error, however, it requires the independence of random error. By employing the chi-square test of homogeneity, whether different columns (or rows) of data in a given table originated from the same population, thereby determining if the observed differences can be solely attributed to sampling error, can be ascertained. Throughout the entire statistical analysis process, the significance level was rigorously set at *p* < 0.05, ensuring the reliability and accuracy of the results.

For the meticulous statistical analysis of the application data, a combination of Microsoft Excel and Google Colab with Jupyter Notebooks provided the necessary computational tools. In addition, several essential libraries, namely scikit-learn 0.22.2.post1, Pandas v0.25.3 and Matplotlib Python v3.2.0, were employed to facilitate the processing and visualisation of the data. 

To optimise the dataset’s handling and interpretation, we decided to employ principal component analysis (PCA) with varimax rotation. This data reduction technique effectively reduced the dataset’s dimensionality while preserving the crucial aspects of interpretability and minimising the loss of vital information. The process involved preprocessing, normalisation and principal component calculation, all performed using the versatile scikit-learn 1.2.1 library. Moreover, we narrowed our focus to specific food attributes, including energy content, proteins, carbohydrates, sugar, total fat, saturated fat, fibre and sodium/salt, ensuring a comprehensive yet targeted analysis of the dataset. The relative importance of each nutrient was calculated and the result was a two-dimension plot where global differences may be visualised as two separate dispersion groups for the two categories.

## 3. Results

### 3.1. Description of the Sample

As shown in Table 1 and Table 2, a total of 2371 processed foods were analysed, which were classified into seven specific types. Six of them were cereal-based meat analogues and one was a plant-based meat analogue. A notable proportion of foods bore nutrition claims (NCs) on fibre (27.2%). The prevalence ranged from 34.7% in breakfast cereals to 18% in cereal cakes/crackers. Interestingly, ‘high fibre’ was the most frequently found claim in the whole sample, being present in five of the seven food types. By contrast, ‘source of fibre’ was 3- to 5-fold more prevalent in two food types (cereal cakes/crackers and plant-based meat analogues).

### 3.2. Nutrient Composition

Foods were classified into two categories: those with fibre-related NCs and those without. Both categories were compared and the nutrient composition is shown in Table 3. Many statistically significant differences were observed. As may be expected, fibre content was higher in all food types with fibre-related NCs (35–103% increase). Biscuits bearing these NCs presented positive differences in all nutrients and energy, except sodium, which was higher. Bread followed with divergences in all nutrients and energy, except sugar. Three of the changes may be considered negative (less protein, more total and saturated fat). In contrast, bars and cereal cakes/crackers with fibre-related NCs only displayed higher fibre and lower carbohydrate median values. Only 3–4 differences in each of them were observed in the other three food types, several of which were negative (greater total fat content in breakfast cereals and toasted bread and similar).

Despite all these statistically significant differences, only a few of them may be considered nutritionally relevant (see Material and Methods). The main difference was the increased fibre content in all food types, already described above. Biscuits and toasted bread and similar bearing fibre-related NCs had lower values for saturated fat, while total fat content was higher in bread and plant-based meat analogues. No other nutritionally relevant difference was observed.

**Figure 1:**
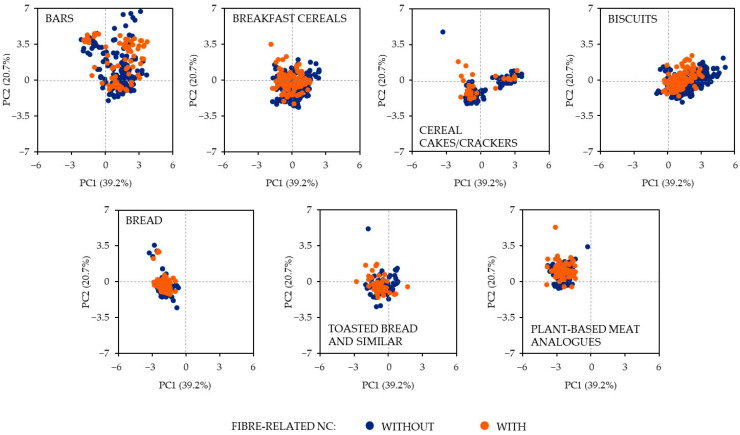
Principal Component Analysis (PCA) based on the nutrient composition of products included in this study, by food type. Nutrients considered (in 100 g or 100 mL): energy (kcal), proteins (g), carbohydrates (g), sugar (g), total fat (g), saturated fat (g), fibre (g) and sodium (g). Processed foods without fibre-related NC; processed foods with these claims.

The consequence of this nutrient composition analysis is that the two categories are indistinguishable when nutrients and energy are plotted, by food type, in a two-dimension graph (two principal components analysis, PCA) (Figure 1). The only exception is a slight shift to the right for biscuits without fibre-related NCs, as a result of all the statistically significant differences observed in Table 3. The two principal components calculated explained 59.9% of the total variability among products, with 39.2% and 20.7% for PC1 and PC2, respectively. 

The greater fibre content in foods with NCs on this nutrient suggested a more frequent use of whole grain cereals and fibre-specific ingredients (see Material and Methods). This hypothesis was confirmed for most food types (Table 4). In fact, at least 50% of six of the seven food types with fibre-related NCs contained whole grain cereals or fibre-specific ingredients. It is noteworthy that a significant proportion of foods without those claims also used whole grain cereals or fibre-specific ingredients quite frequently (27.7% and 22.6%, respectively).

### 3.3. Nutritional Quality

Whether the changes observed in nutrient composition are correlated with alterations in the nutritional quality was explored next. For this purpose, the Nutrient Profile Model developed by the Pan American Health Organization was used (PAHO-NPM) [36]. The results indicate that as many as 88.7% of processed foods bearing fibre-related NCs were ‘less healthy’ (Table 5). Sodium was the condition with the highest proportion of items exceeding the threshold (43.4%), followed by total fat (43.1%) and free sugar (40.4%). When analysed by food type, some concerning results were observed. The proportion of ‘less healthy’ items was greater than 90% for four of the seven food types. Specifically, 98.1% of bread, 97.5% of plant-based meat analogues and 81.1% of toasted bread and similar were high in sodium. Plant-based analogue was the food type with the highest proportion of items exceeding the threshold for fat (88.9%), followed by biscuits (85.1%). Biscuits and bars had the highest percentage of foods high in free sugar (76.7% and 72.1%, respectively). In addition, most bars were also high in saturated fat (71.8%). 

The two categories were compared next and no differences were observed in the proportion of foods considered ‘less healthy’ in the whole sample or by food type (Table 5). When analysing by nutrient, foods with fibre-related NCs had a lower proportion of items high in fat, free sugar and saturated fat. On the contrary, sweeteners (LNCS) were more frequently used, mostly due to a three-fold greater presence in biscuits. 

Food types were also individually studied and eight specific divergences were observed, most of them indicating a deterioration of the nutritional quality (Table 5). In fact, only three of them were improvements: a lower percentage of toasted bread and similar with fibre-related NCs was high in fat, while fewer biscuits were high in free sugar and saturated fat. On the contrary, more bars were high in saturated fat among those with fibre-related NCs. In addition, the proportion of biscuits high in sodium and with LNCS was greater and more bread and plant-based meat analogues were high in fat. 

### 3.4. Micronutrient Fortification and Organic Foods

As mentioned in the Introduction, micronutrient fortification may potentiate the positive perception of processed foods bearing fibre-related NCs. When mineral and vitamin fortification was analysed, no differences were observed compared to foods without these claims, either in the whole sample or by food type (Table 6). Nevertheless, it is interesting to note that bars are the food type with the highest fortification rate. 

Organic products may also draw consumers’ attention to products with fibre-related NCs. However, those foods presented a lower prevalence of the organic version in this study (Table 6). This was also the case for the three food types presenting statistically significant differences.

## 4. Discussion

The main conclusion of this work is that processed foods with fibre-related NCs are not generally healthy nor better than the rest. The use of the PAHO-NPM resulted in as many as 88.7% of them being considered less healthy. In addition, they did not differ from those without these NCs. Individually, there are differences in specific critical nutrients and food types. Contrary to expectations, most of them indicate a deterioration of the nutritional quality in foods bearing fibre-related NCs. Regarding nutrient composition, all food types with these claims contained a higher amount of fibre. As for the rest of the nutrients, only a few nutritionally relevant differences were observed and only half of them were improvements. 

Processed foods with fibre-related NCs more frequently used whole grain cereals and other specific ingredients to increase their fibre content. Regarding other strategies to improve consumers’ acceptance, they did not present higher rates of vitamin and mineral fortification. However, the prevalence of the organic version was lower among foods with fibre-related NCs. 

### 4.1. Fibre-Related NCs

In the present work, 27.1% of all foods bore an NC on fibre. This result cannot be compared with preceding studies because food types were selected for their high use of these NCs, following Ropero et al., 2023 [7]. In addition, only the main food image provided by the manufacturer or the online supermarket was checked for NCs in this study. Those located elsewhere on the package or on the websites were not registered and, therefore, the real prevalence of NCs may be underrepresented. 

Nevertheless, the frequency of these NCs can be compared in specific food types. NCs on fibre are virtually absent in foods from animal origin, while their use in plant-based foods is variable [7]. A work performed in Italy by Martini et al. obtained that 48% of 376 breakfast cereals presented these kinds of NCs [14]. These results displayed higher values than the ones obtained in this study with a slightly larger sample (34.4%, 421 items). 

As many as 31.8% of breads presented fibre-related NCs in this study, which is similar to the results in pre-packed bread in Lebanon (29.3%) [9]. An Irish study showed practically identical prevalence (30.3%), though bakery products were included along with bread [11].

The use of NCs on fibre in plant-based meat analogues is quite interesting because this nutrient is absent in meat. Therefore, these NCs may be a way for these foods to stand out from meat. In fact, as many as 28.2% of 285 of these products bore fibre-related NCs in the present study. A recent Italian work reported a slightly higher prevalence (34.5%, 229 foods), while a considerably lower rate was observed in the USA (10.6%, 216 items) [10,13].

The present results show that ‘high fibre’ is more frequently used than ‘source of fibre’ in five of the seven food types. Martini et al. obtained a similar outcome in breakfast cereals [14]. This is surprising because the amount of fibre required to use ‘high fibre’ is doubled and fewer foods should meet this condition [35]. 

### 4.2. Nutrient Composition and Nutritional Quality

The present results show that most processed foods having at least one fibre-related NC were considered ‘less healthy’ according to the PAHO-NPM. No differences were observed between the two categories studied. As a consequence, the two analysed categories contribute equally to unhealthy diets. An unhealthy diet is one of the four main risk factors for developing non-communicable diseases (NCDs) [40]. According to the World Health Organization (WHO), ‘NCDs kill 41 million people each year, equivalent to 74% of all deaths’ [40]. Therefore, most processed foods included in this work contribute to this burden, regardless of the presence of fibre-related NCs. 

An important proportion of processed foods bearing NCs on fibre exceeded the thresholds to be considered high in sodium, total fat and free sugar. The 14% reduction in the percentage of items high in free sugar compared to those without these claims, though significant, is of little relevance. According to a report issued in 2015 by the WHO, ‘a high level of free sugars intake is of concern, because of its association with poor dietary quality, obesity and risk of NCDs’ [41]. In 2022, the European Food and Safety Authority (EFSA) advocated an intake of free sugars that is as low as possible [42]. Free sugar intake in adults in Europe is quite high considering this recommendation (7–17% of total energy) [43]. The high number of processed foods bearing fibre-related NCs that are high in free sugar contributes to this excess. This is particularly important because these NCs may produce positive attitudes among consumers and they may be unaware of the presence of high free sugar and its health risks [17,21,22]. 

Nearly all bread and plant-based meat analogues are high in sodium. Around three quarters of the total salt intake comes from processed foods and meals prepared outside the home [44]. According to the World Health Organization (WHO), most people consume around twice the recommended maximum (salt) intake [45]. This institution also acknowledges that, ‘reducing sodium intake is one of the most cost-effective ways to improve health and reduce the burden of noncommunicable diseases’ [46]. In addition, high sodium intake has negative effects on blood pressure levels, hypertension and cardiovascular disease [44,47]. For all these reasons, the WHO advocates a 30% reduction in sodium intake by 2030 as the main dietary factor to decrease the incidence of NCDs [40]. To contribute to this goal, a high proportion of processed foods analysed in the present study should be reformulated to reduce their sodium content, regardless of the presence of fibre-related NCs. 

Despite the lower number of foods high in saturated fat, one in five with fibre-related NCs exceeded the threshold. The proportion is particularly large for bars (71.8%). According to the recent WHO guidelines, ‘higher dietary intakes of saturated fat were associated with increased mortality’ [48]. Therefore, a further reduction in the proportion of foods that are high in saturated fat is highly desirable. 

Interestingly, the prevalence of any kind of sweetener (LNCS) is much higher among processed foods with fibre-related NCs. It may be a way to reduce sugar content and become more appealing to consumers while maintaining sweetness. However, consumers’ perceptions of LNCS are not generally positive and its addition to these foods may deteriorate their good opinion of them [49]. In addition, some international institutions have not recommended LNCS for several years. This is the case of the Pan American Health Organization and the WHO Regional Offices for Europe and the Americas [36,50]. Recently, the WHO has released a new report on non-sugar sweeteners (NSSs; polyols excluded) [51]. The WHO ‘suggests that non-sugar sweeteners not be used as a means of achieving weight control or reducing the risk of NCD’ [51]. Additionally, the report states that, ‘long-term NSS use was associated with increased risk of type 2 diabetes, cardiovascular diseases and mortality’ [51]. Thus, the addition of LNCS to attract consumers’ interest in foods with fibre-related NCs may be detrimental. 

Regarding nutrient composition, interpretation of data in this study or those of other authors must be cautious. The main reason is that small changes in any nutrient or energy are expected to have little or no practical impact on the diet of the general population. Accordingly, in this work relevant criteria were applied in order to determine whether differences are nutritionally relevant following the European Regulation (EC) No 1924/2006 and previously published work (see Material and Methods) [31,35]. To be coherent with this, the review of previous evidence follows the same criteria. 

The most striking difference observed in the present work was a greater fibre content in processed foods with fibre-related NCs compared to those without. The more frequent use of whole grain cereals or any fibre-specific ingredient observed may contribute to this. 

So far, only three publications have investigated potential nutritional differences between products bearing fibre-related NCs and those without. A work in Canada analysed 15,184 foods of all types and those with fibre-related NCs presented more than double the proportion of ‘less healthy’ items [12]. Results presented here do not agree with these findings, since no differences were observed in the proportion of ‘less healthy’ foods. Two important reasons may be responsible for this divergence. One is that only seven specific food types were analysed. The other reason is that they used a different NPM and results may vary considerably depending on the used NPM, as previously demonstrated [33,52].

In the second publication, Martini et al. analysed 376 breakfast cereals available on the Italian market [14]. Those claiming to be a ‘source of fibre’ only differed in this nutrient. On the contrary, foods claiming to be ‘high in fibre’ diverged in energy and all nutrients tested, except saturated fat. However, only differences in total fat, fibre and salt were nutritionally relevant (higher/lower than 30%; 25% for salt). In the present work, only the increase in fibre content was nutritionally significant, maybe because all foods with any NC on fibre were pooled together. 

Finally, Cutroneo et al. studied 229 plant-based meat analogues and obtained that those with fibre-related NCs presented statistically significant increases in energy content and four nutrients [13]. However, only three of them were nutritionally relevant (more carbohydrates, sugar and fibre). The present results show that only increments in fibre and total fat were nutritionally significant. 

It would be expected that the evaluation of the nutrient composition and the use of a nutrient profile model would produce similar results. Therefore, given the few differences in the former, few were expected in the latter. However, interesting divergences were observed, particularly because most of them reflected worse nutritional quality. 

### 4.3. Nutrient Fortification and Organic Origin

Nutrient fortification may be an extra mechanism to entice consumers to purchase foods with fibre-related NCs [24,25]. It is interesting that results in the present work show that foods with fibre-related NCs do not use micronutrient fortification more frequently. It can be speculated that manufacturers are likely to consider those NCs sufficient to draw consumers’ attention and no further claims are needed. 

The same concept applies to organic foods. In fact, processed foods with fibre-related NCs presented lower prevalence of the organic version. To our knowledge, only one previous work has studied this, with similar results. Martini et al. observed that the frequency of organic breakfast cereals with fibre-related NCs was lower than those without them [14]. The reduction was smaller than in the present study (12%, while 26% in the present study) and no statistics were applied to determine its significance.

### 4.4. Strenghts and Limitations

Some of the important strengths in the present work are listed as follows:

- This is the first exhaustive research study of seven specific processed food types bearing fibre-related NCs, analysing both their nutrient composition and their healthiness by applying an NPM;
- This is the first published paper comparing the nutrient composition and healthiness of processed foods with fibre-related NCs and those without;
- This is the first report comparing processed foods with fibre-related NCs and those without in the Spanish market;
- This is also the first manuscript describing the micronutrient fortification of processed foods with fibre-related NCs and comparing them with those without these claims;
- The food types included in this analysis were selected for their high prevalence of fibre-related NCs according to Ropero et al., 2023 [31];
- The sample by food type and category is significant;
- Data were collected several years after European Regulation (EC) No 1924/2006 on nutrition claims was fully in force [35];
- Nonetheless, the present work has a few important limitations:
- Selection of brands did not follow criteria based on customers’ purchases or the most popular products;
- Data collected were reliant on the accuracy of the information provided on the manufacturers’ and supermarkets’ websites;
- Some of the products displayed 0 g salt/sodium, which could be wrongly rounded, despite the non-compulsory guidance published by the EC [53];
- Only the main food image was used in the NC recording process in order to maintain rigour throughout the sample;
- According to Regulation (EU) 1169/2011, it is not compulsory to display fibre content in the nutrient declaration [34]. Therefore, fibre content was missing in some of the analysed foods.

## 5. Conclusions

The main conclusion of this work is that consumers’ positive perception regarding foods with fibre-related NCs is incorrect. Results show that these foods are not better than those without these claims and their nutritional quality is somewhat worse. In fact, most processed foods with NCs on fibre are ‘less healthy’. Consumers are unaware of the high presence of nutrients negatively associated with health and this poses a risk to them. 

## Figures and Tables

**Table 1:** Description of the food types included in the study.

Types	Foods
Bars	Bars made of cereals, legumes, fruits or nuts with or without added ingredients
Biscuits	All kinds of biscuits according to their commercial name, including wafers. Savoury biscuits were excluded
Bread	Bread (soft) and similar products made with yeast
Breakfast cereals	Flakes, muesli, granola, extruded, ready-to-eat cereals
Cereal cakes/crackers	Cereal cakes and crackers with no yeast or gasifiers added
Plant-based meat analogues	Any product made to resemble meat and made with plant ingredients
Toasted bread and similar	Toasted bread and similar products made with yeast (low water content)

**Table 2:** Items included in the study and presence of fibre-related NCs.

Food Types	Total	No. Foods with NC-Fibre (%)	No. NCs	
			Source of Fibre (%) *	High Fibre (%) *
Bars	270	88 (32.6)	40 (44.9)	49 (55.1)
Biscuits	638	134 (21)	54 (40.3)	80 (59.7)
Bread ^1^	340	108 (31.8)	39 (36.1)	68 (63)
Breakfast cereals ^2^	421	146 (34.7)	52 (35.1)	96 (64.9)
Cereal cakes/crackers	178	32 (18)	25 (78.1)	7 (21.9)
Plant-based meat analogues	285	81 (28.4)	68 (84)	13 (16)
Toasted bread and similar	239	55 (23)	25 (43.9)	32 (56.1)
Total	2371	644 (27.2)	303 (46.7)	345 (53.2)

NC-Fibre: fibre-related NC; %: percentage within the food type or the total sample (Total); *: percentage of the total NCs on fibre; ^1^: one item bears the NC ‘more fibre’; ^2^ two items bore two fibre-related NCs.

**Table 3:** Energy and nutrient density of specific food types. Values in 100 g or 100 mL.

Food Types	NC-Fibre	Energy (kcal)			Protein (g)			Carbohydrates (g)			Sugar (g)		
		n	Median (IR)	*p*-Value	n	Median (IR)	*p*-Value	n	Median (IR)	*p*-Value	n	Median (IR)	*p*-Value
Bars	No	182	426 (379; 468)	0.719	182	10.2 (6.6; 27)	0.819	182	50 (38; 61.8)	<0.05 *	180	25.5 (13.9; 34)	0.165
	Yes	88	402 (360; 511)		88	13.4 (6.3; 23)		87	38 (24; 64.5)		88	22 (12.5; 28.2)	
Biscuits	No	504	477 (455; 500)	<0.001 *	504	6.4 (5.5; 7.2)	<0.001 *	504	65.7 (62; 70)	<0.001 *	499	25 (20; 33)	<0.001 *
	Yes	134	459 (442; 471)		134	7.2 (6.3; 8)		134	63 (59; 67)		133	19 (15; 23)	
Bread	No	232	263 (246; 278)	<0.01 *	232	9.3 (8.3; 10)	<0.001 *	232	46 (42.1; 50)	<0.001 *	222	3.7 (2.1; 5)	0.497
	Yes	108	250 (228; 273)		108	6.8 (3.9; 9.7)		108	41 (35; 46)		100	3.5 (2.2; 4.7)	
Breakfast cereals	No	275	380 (368; 422)	0.665	275	9.2 (7.5; 11.3)	<0.001 *	275	67.9 (61; 77.2)	<0.001 *	272	16 (2.2; 23.2)	0.053
	Yes	146	381 (367; 406)		146	10.6 (9; 12)		146	64 (58; 69)		145	13 (3.5; 18)	
Cereal cakes/crackers	No	146	389 (380; 458)	0.416	146	7.6 (7; 8.5)	0.186	146	76.3 (70; 81)	<0.001 *	145	1.5 (0.7; 19)	0.771
	Yes	32	389 (377; 456)		32	8.1 (7; 10.1)		32	70 (64; 75.6)		32	2 (0.5; 21.3)	
Plant-based meat analogues	No	201	204 (179; 237)	0.159	204	14.1 (7.7; 18.2)	0.544	201	12.7 (7.3; 18)	0.09	204	1.8 (0.9; 2.7)	<0.05 *
	Yes	81	219 (187; 246)		81	12 (7.2; 17)		81	9.5 (5; 18)		81	1.4 (0.7; 2.1)	
Toasted bread and similar	No	184	420 (396; 456)	<0.001 *	182	12 (9.5; 13)	0.629	183	66 (60; 70)	0.545	182	3 (1.8; 4.5)	0.234
	Yes	55	406 (387; 422)		55	11.1 (10.5; 13)		55	64 (60; 69)		55	3.3 (2; 4.9)	
**Food types**	**NC-Fibre**	**Total fat (g)**			**Saturated fat (g)**			**Fibre (g)**			**Sodium (mg)**		
		**n**	**Median (IR)**	***p*-value**	**n**	**Median (IR)**	***p*-value**	**n**	**Median (IR)**	***p*-value**	**n**	**Median (IR)**	***p*-value**
Bars	No	182	15.6 (12; 22)	0.898	180	5.2 (2.7; 9.6)	0.791	156	6 (4.1; 7.5)	<0.001 *	181	116 (32; 204)	0.241
	Yes	88	13.6 (10; 33.3)		85	5.1 (4.1; 8)		83	9.9 (7.3; 14)		87	92 (50; 178)	
Biscuits	No	504	20 (16; 24)	<0.01 *	503	7.6 (2.9; 13)	<0.001 *	384	3.2 (2.3; 4.9)	<0.001 *	504	232 (160; 320)	<0.05 *
	Yes	134	19 (16; 21)		134	3.2 (1.7; 7)		133	6.5 (5; 8)		134	280 (154; 380)	
Bread	No	232	3.8 (2.6; 5.1)	<0.001 *	214	0.7 (0.5; 1)	<0.05 *	175	3.6 (2.9; 6.1)	<0.001 *	223	480 (400; 520)	<0.05 *
	Yes	107	5 (3.3; 6.9)		103	0.8 (0.6; 1.1)		102	6.8 (5.8; 9)		107	424 (396; 480)	
Breakfast cereals	No	275	5.3 (2.5; 12)	<0.05 *	272	1.2 (0.6; 2.7)	0.131	259	6.4 (3.9; 9)	<0.001 *	270	82 (8; 252)	0.6
	Yes	146	6.8 (3.9; 11)		145	1.3 (0.8; 2.4)		143	8.9 (6.7; 10.9)		144	68 (12; 177)	
Cereal cakes/crackers	No	146	3.3 (2.2; 17)	0.371	146	0.8 (0.5; 9)	0.475	126	3 (2; 4.3)	<0.001 *	146	234 (61; 440)	0.381
	Yes	31	4.6 (2.5; 17.5)		31	0.8 (0.6; 10.5)		31	4.8 (4; 7.3)		32	288 (195; 410)	
Plant-based meat analogues	No	202	9.8 (6.2; 15)	<0.05 *	202	1.3 (1; 1.8)	0.115	152	3.1 (2.1; 4.6)	<0.001 *	204	520 (440; 640)	0.466
	Yes	81	13 (8.3; 15.7)		81	1.3 (1; 2.4)		77	5 (4; 6.2)		80	510 (406; 600)	
Toasted bread and similar	No	184	11 (6.7; 17.3)	<0.01 *	174	1.9 (1; 3.1)	<0.01 *	150	4.5 (3.7; 7.2)	<0.001 *	184	634 (520; 760)	0.164
	Yes	55	9.5 (6.6; 11)		55	1.2 (0.8; 1.9)		53	7.9 (5; 10)		55	560 (400; 720)	

NC-Fibre: fibre-related NC; IR: interquartile range; *: statistically significant differences according to *p* < 0.05; n: foods with data.

**Table 4:** Foods with whole grain cereal and with fibre-specific ingredients, by food type.

Food Types	NC-Fibre	Foods with Whole Grain Cereal			Foods with Fibre-Specific Ingredients		
		n	No (%)	*p*-Value	n	No (%)	*p*-Value
Bars	No	176	29 (16.5)	0.268	176	49 (27.8)	<0.001 *
	Yes	87	20 (23)		87	50 (57.5)	
Biscuits	No	504	84 (16.7)	<0.001 *	504	149 (29.6)	<0.001 *
	Yes	134	82 (61.2)		134	84 (62.7)	
Bread	No	231	73 (31.6)	<0.01 *	231	70 (30.3)	<0.001 *
	Yes	107	52 (48.6)		107	76 (71)	
Breakfast cereals	No	275	126 (45.8)	<0.01 *	275	24 (8.7)	<0.001 *
	Yes	146	88 (60.3)		146	36 (24.7)	
Cereal cakes/crackers	No	146	71 (48.6)	0.76	146	2 (1.4)	<0.001 *
	Yes	32	14 (43.8)		32	6 (18.8)	
Plant-based meat analogues	No	199	34 (17.1)	0.195	199	73 (36.7)	<0.01 *
	Yes	81	20 (24.7)		81	45 (55.6)	
Toasted bread and similar	No	179	56 (31.3)	<0.05 *	179	20 (11.2)	0.906
	Yes	54	27 (50)		54	7 (13)	
Total	No	1710	473 (27.7)	<0.001 *	1710	387 (22.6)	<0.001 *
	Yes	641	303 (47.3)		641	304 (47.4)	

NC-Fibre: fibre-related NC; %: percentage within the food type; n: foods with data; *: statistically significant differences according to *p* < 0.05.

**Table 5:** Classification of foods as high in critical nutrients according to the PAHO-NPM [36], by food subtype.

Food Types	NC-Fibre	‘Less Healthy’			High Fat			High Free Sugar			High Saturated Fat			High Sodium			Sweeteners (LNCS)		
		n	No (%)	*p*-Value	n	No (%)	*p*-Value	n	No (%)	*p*-Value	n	No (%)	*p*-Value	n	No (%)	*p*-Value	n	No (%)	*p*-Value
Bars	No	179	172 (96.1)	0.811	182	112 (61.5)	0.136	180	136 (75.6)	0.649	180	98 (54.4)	<0.05 *	181	3 (1.7)	0.557	182	57 (31.3)	0.216
	Yes	82	80 (97.6)		88	45 (51.1)		86	62 (72.1)		85	61 (71.8)		87	0 (0)		88	35 (39.8)	
Biscuits	No	498	495 (99.4)	1	504	408 (81)	0.33	499	442 (88.6)	<0.001 *	503	315 (62.6)	<0.001 *	504	40 (7.9)	<0.05 *	504	33 (6.5)	<0.001 *
	Yes	133	132 (99.2)		134	114 (85.1)		133	102 (76.7)		134	43 (32.1)		134	20 (14.9)		134	26 (19.4)	
Bread	No	205	193 (94.1)	0.101	232	10 (4.3)	<0.05 *	222	10 (4.5)	0.742	214	3 (1.4)	1	223	209 (93.7)	0.141	232	0 (0)	0.695
	Yes	98	97 (99)		107	13 (12.1)		100	3 (3)		103	2 (1.9)		107	105 (98.1)		108	1 (0.9)	
Breakfast cereals	No	264	184 (69.7)	0.319	271	50 (18.5)	0.173	273	156 (57.1)	0.539	268	23 (8.6)	0.738	266	25 (9.4)	0.804	275	6 (2.2)	0.837
	Yes	140	90 (64.3)		142	18 (12.7)		144	77 (53.5)		141	10 (7.1)		140	15 (10.7)		146	2 (1.4)	
Cereal cakes/crackers	No	144	93 (64.6)	0.414	144	40 (27.8)	1	144	43 (29.9)	1	144	43 (29.9)	0.962	144	46 (31.9)	0.391	146	4 (2.7)	1
	Yes	31	23 (74.2)		31	9 (29)		31	9 (29)		31	10 (32.3)		31	13 (41.9)		32	1 (3.1)	
Plant-based meat analogues	No	199	192 (96.5)	0.519	201	151 (75.1)	<0.05 *	199	2 (1)	0.902	201	25 (12.4)	0.381	201	190 (94.5)	0.439	204	0 (0)	1
	Yes	81	80 (98.8)		81	72 (88.9)		81	0 (0)		81	14 (17.3)		81	79 (97.5)		81	0 (0)	
Toasted bread and similar	No	173	164 (94.8)	0.093	184	62 (33.7)	<0.001 *	183	5 (2.7)	1	174	18 (10.3)	0.736	184	161 (87.5)	0.34	183	0 (0)	0.527
	Yes	53	46 (86.8)		53	3 (5.7)		53	1 (1.9)		53	4 (7.5)		53	43 (81.1)		55	1 (1.8)	
Total	No	1662	1493 (89.8)	0.468	1718	833 (48.5)	<0.05 *	1700	794 (46.7)	<0.01 *	1684	525 (31.2)	<0.001 *	1703	674 (39.6)	0.1	1726	100 (5.8)	<0.001 *
	Yes	618	548 (88.7)		636	274 (43.1)		628	254 (40.4)		628	144 (22.9)		633	275 (43.4)		644	66 (10.2)	

%: percentage within the food type; n: foods with data; *: statistically significant differences according to *p* < 0.05; thresholds used to consider foods as high in critical nutrients are [36]: ≥30% of total energy from total fat, ≥10% of total energy from free sugars, ≥10% of total energy from saturated fat, ≥1 mg sodium/kcal; No: foods exceeding the threshold or with LNCS; LNCS: low- and no-calorie sweetener.

**Table 6:** Foods fortified with minerals or vitamins and organic prevalence, by food type.

Food Types	NC-Fibre	Foods with Added Minerals			Foods with Added Vitamins			Organic Foods		
		n	No (%)	*p*-Value	n	No (%)	*p*-Value	n	No (%)	*p*-Value
Bars	No	176	17 (9.7)	0.599	176	34 (19.3)	0.349	182	40 (22)	<0.01 *
	Yes	87	11 (12.6)		87	12 (13.8)		88	5 (5.7)	
Biscuits	No	504	31 (6.2)	0.843	504	39 (7.7)	0.417	504	131 (26)	0.699
	Yes	134	7 (5.2)		134	7 (5.2)		134	32 (23.9)	
Bread	No	231	7 (3)	0.225	231	5 (2.2)	0.294	232	41 (17.7)	0.398
	Yes	107	7 (6.5)		107	0 (0)		108	24 (22.2)	
Breakfast cereals	No	275	24 (8.7)	0.202	275	30 (10.9)	0.054	275	161 (58.5)	<0.001 *
	Yes	146	7 (4.8)		146	7 (4.8)		146	55 (37.7)	
Cereal cakes/crackers	No	146	8 (5.5)	1	146	9 (6.2)	1	146	71 (48.6)	0.204
	Yes	32	2 (6.3)		32	2 (6.3)		32	11 (34.4)	
Plant-based meat analogues	No	203	12 (5.9)	0.843	203	16 (7.9)	0.757	204	79 (38.7)	<0.001 *
	Yes	81	6 (7.4)		81	8 (9.9)		81	12 (14.8)	
Toasted bread and similar	No	179	2 (1.1)	1	179	2 (1.1)	1	184	41 (22.3)	0.39
	Yes	54	0 (0)		54	0 (0)		55	16 (29.1)	
Total	No	1714	101 (5.9)	0.827	1714	135 (7.9)	0.073	1727	564 (32.7)	<0.001 *
	Yes	641	40 (6.2)		641	36 (5.6)		644	155 (24.1)	

NC-Fibre: fibre-related NC; %: percentage within the food type; n: foods with data; *: statistically significant differences according to *p* < 0.05.

## Data Availability

Data used in this work are available online at https://badali.umh.es.
